# Supplementary material for: LncRNA MIR200CHG inhibits EMT in gastric cancer by stabilizing miR-200c from target-directed miRNA degradation
Source: Nat Commun. 2023 Dec 8;14:8141. doi: 10.1038/s41467-023-43974-w (PMC10709323; doi:10.1038/s41467-023-43974-w)
Supplement: Supplementary file 1 — Supplementary Information [file 41467_2023_43974_MOESM1_ESM.pdf]

Supplementary Information

LncRNA MIR200CHG inhibits EMT in gastric cancer by stabilizing miR-200c from target-directed miRNA degradation

Yixiao Zhu[1,2,3†], Chengmei Huang[1,†], Chao Zhang[1,†], Yi Zhou[1,†], Enen Zhao[1], Yaxin Zhang[1], Xingyan Pan[1], Huilin Huang[1,*], Wenting Liao[1,*], Xin Wang[2,4,5*]

[1] *State Key Laboratory of Oncology in South China, Collaborative Innovation Center for Cancer Medicine, Sun Yat-Sen University Cancer Center, Guangzhou, Guangdong, China*

[2] *Department of Surgery, The Chinese University of Hong Kong, Hong Kong SAR, China*

[3] *National Clinical Research Centre for Geriatric Disorders, Department of Geriatrics, Xiangya Hospital, Central South University, Changsha, Hunan, China*

[4] *Li Ka Shing Institute of Health Sciences, The Chinese University of Hong Kong, Hong Kong SAR, China*

[5] *Shenzhen Research Institute, The Chinese University of Hong Kong, Shenzhen, Guangdong, China*

[*] Correspondence: xinwang@cuhk.edu.hk; liaowt@sysucc.org.cn; huanghl1@sysucc.org.cn

[†] Yixiao Zhu, Chengmei Huang, Chao Zhang and Yi Zhou contributed equally to this work.

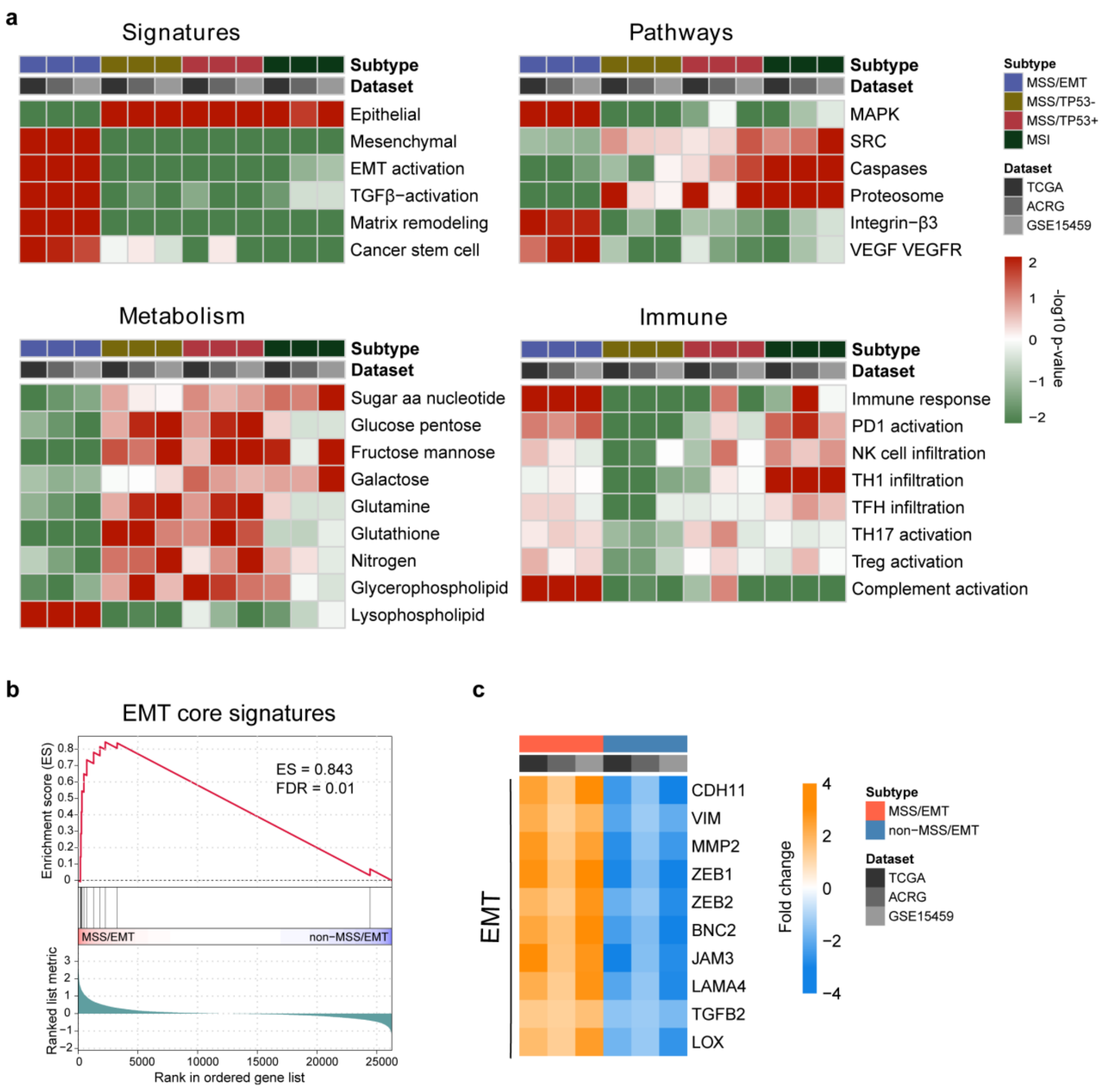

Supplementary Figure 1. An overview of biological characteristics associated with different GC subtypes. a The heat maps illustrate consistent gene set enrichment patterns of biological processes and signaling pathways across three independent datasets (TCGA, *n* = 54 samples for MSS/EMT, *n* = 43 samples for MSS/TP53-, *n* = 44 samples for MSS/TP53+, *n* = 38 samples for MSI; ACRG, *n* = 46 samples for MSS/EMT, *n* = 107 samples for MSS/TP53-, *n* = 79 samples for MSS/TP53+, *n* = 68 samples for MSI; GSE15459, *n* = 83 samples for MSS/EMT, *n* = 61 samples for MSS/TP53-, *n* = 37 samples for MSS/TP53+, *n* = 11 samples for MSI). GSEA was performed based on gene sets of canonical signatures, pathways,

metabolic pathways, and immune cell signatures. Red represents over-enrichment, and green represents under-enrichment, with the color depth proportional to -log10 (*p*-value) calculated based on GSEA. **b** The GSEA plot shows the significant enrichment of the EMT core signature genes in the MSS/EMT subtype of GC in the TCGA dataset (FDR = 0.01, permutation test, *n* = 10,000). ES: enrichment score; FDR: false discovery rate. **c** The heat map shows significant upregulation of representative EMT signature genes in the MSS/EMT subtype compared with the non-MSS/EMT subtypes in all of the three independent datasets (all BH-adjusted $P < 0.0001$).

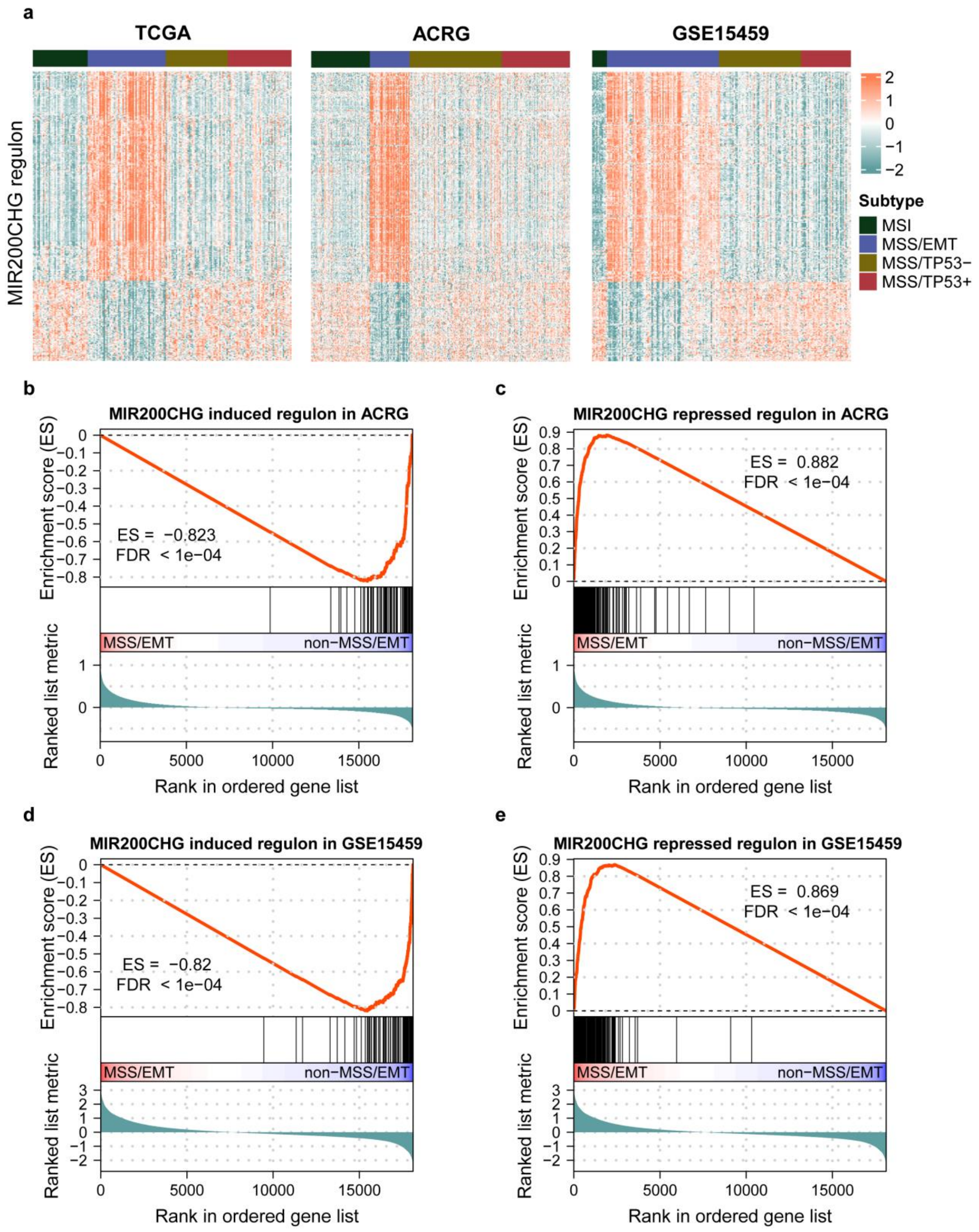

Supplementary Figure 2. The validation of MIR200CHG regulon in the ACRG and GSE15459 datasets. a The heatmaps compare the MIR200CHG regulon expression patterns in the TCGA (*n* = 54 samples for MSS/EMT, *n* = 43 samples for MSS/TP53-, *n* = 44 samples for MSS/TP53+, *n* = 38 samples for MSI), ACRG (*n* = 46 samples for MSS/EMT, *n* = 107 samples for MSS/TP53-, *n* = 79 samples for MSS/TP53+, *n* = 68

samples for MSI) and GSE15459 (*n* = 83 samples for MSS/EMT, *n* = 61 samples for MSS/TP53-, *n* = 37 samples for MSS/TP53+, *n* = 11 samples for MSI) datasets. Rows are sorted by the same order as in TCGA. **b-e** Gene set enrichment plots of MIR200CHG induced and repressed genes in the ACRG and GSE15459 datasets, respectively. The upper panel illustrates the running sum scores of GSEA random walks, the middle and lower panels show the positions of the MIR200CHG induced and repressed genes in the gene list ranked by log2 fold change between MSS/EMT and non-MSS/EMT in the ACRG (**b, c**) and GSE15459 (**d, e**) datasets.

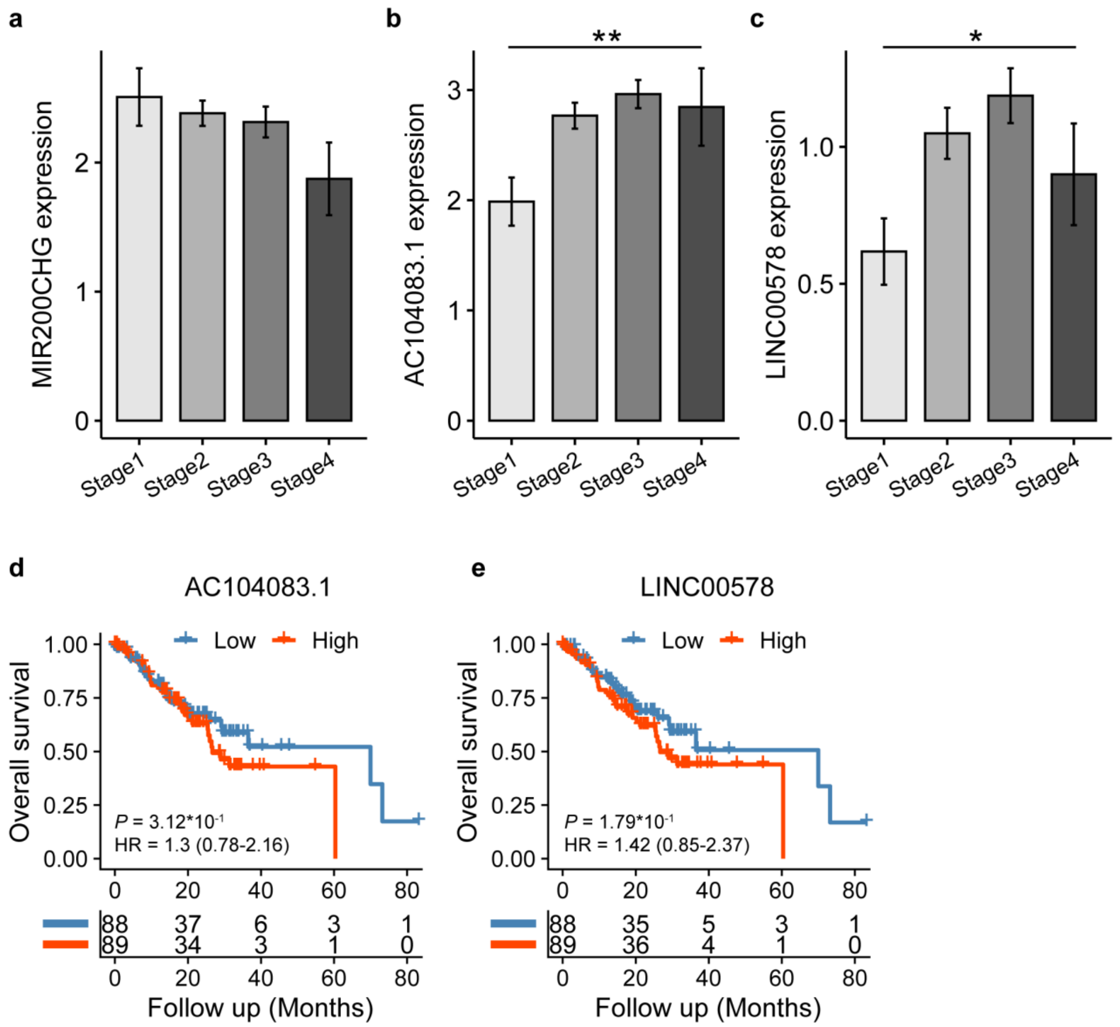

Supplementary Figure 3. Clinical associations of the three master regulatory lncRNAs. a-c The bar plots show a gradual increase or decrease in the expression of MIR200CHG (**a**), AC104083.1 (**b**) and LINC00578 (**c**) in more advanced tumor stages in the TCGA cohort (*n* = 26 samples for Stage 1, *n* = 70 samples for Stage 2, *n* = 70 samples for Stage 3, *n* = 11 samples for Stage 4). *P*-values were calculated by one-way ANOVA (*: $P < 0.05$, **: $P < 0.01$). Each bar in bar plots represents the mean ± standard deviation. **d-e** The KM plots show the survival associations of AC104083.1 (**d**, *P* = 0.31, log-rank test) and LINC00578 (**e**, *P* = 0.18, log-rank test) in the TCGA cohort (*n* = 177 samples). Patients were divided into high- and low-expression subgroups based on the median levels.

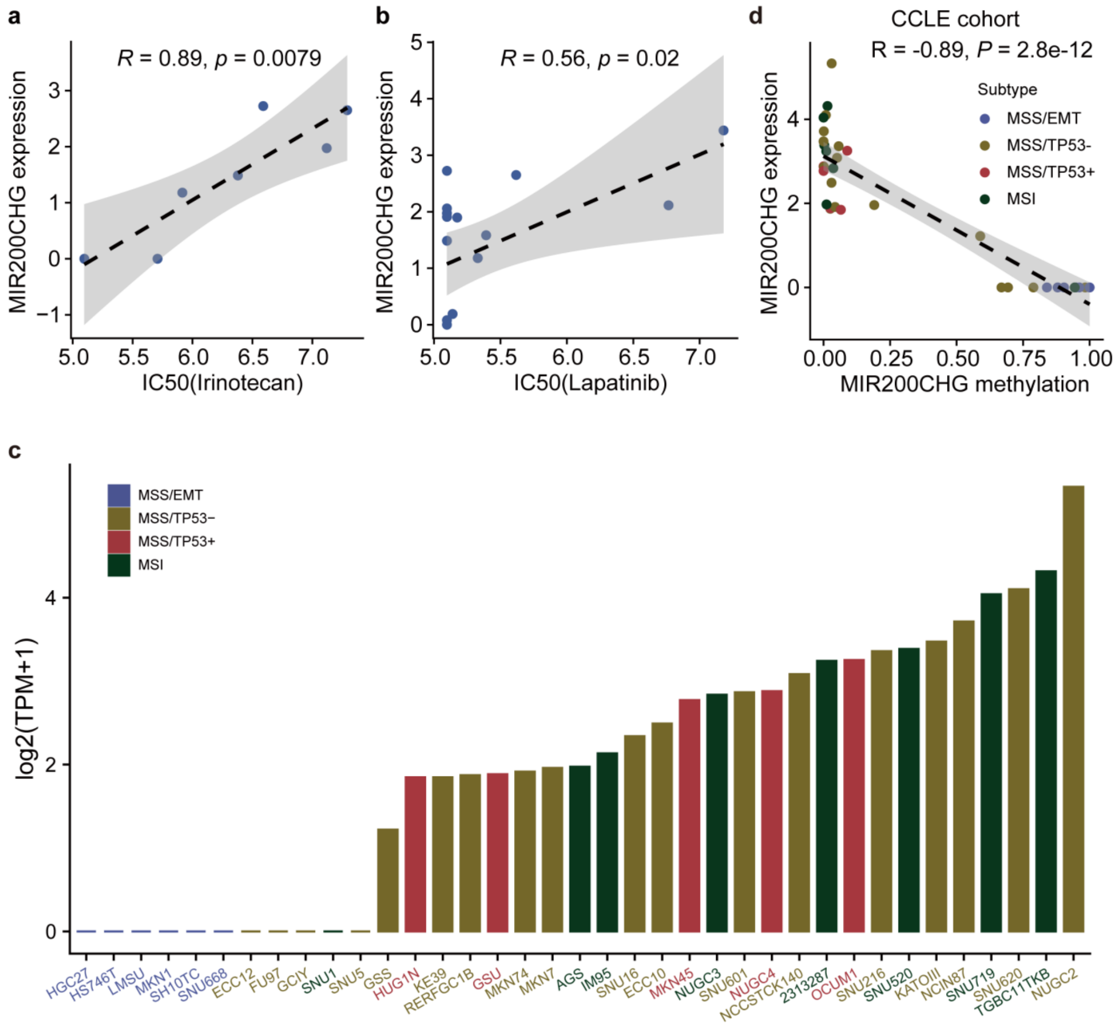

e

Analysis of the coding potential

Transcript name	RNA size (nt)	Coding probability	Coding	Status
MIR200CHG	350	0.020	no	non-coding
UCA1	2299	0.002	no	non-coding
GAPDH	1875	0.999	yes	coding

Supplementary Figure 4. The validation and analysis of MIR200CHG in the CCLE GC cell lines. a-b The scatter plots show the significant associations between MIR200CHG expression and the sensitivities to Irinotecan (**a**) and Lapatinib (**b**) in 18 GC cell lines. **c** The waterfall plot compares the MIR200CHG expression levels in 37

GC cell lines encompassing all the four GC subtypes (*n* = 6 cell lines for MSS/EMT, *n* = 18 cell lines for MSS/TP53-, *n* = 5 cell lines for MSS/TP53+, *n* = 8 cell lines for MSI). **d** The scatter plot confirms the significant inverse correlation between MIR200CHG promoter methylation and expression in 34 GC cell lines (*n* = 6 cell lines for MSS/EMT, *n* = 16 cell lines for MSS/TP53-, *n* = 5 cell lines for MSS/TP53+, *n* = 7 cell lines for MSI). **e** The coding potential of MIR200CHG predicted by the Coding Potential Assessment Tool. The lncRNA UCA1 and the protein-coding gene GAPDH are shown as controls. *P*-values were based on two-sided Pearson's correlation tests **(a, b, d)**.

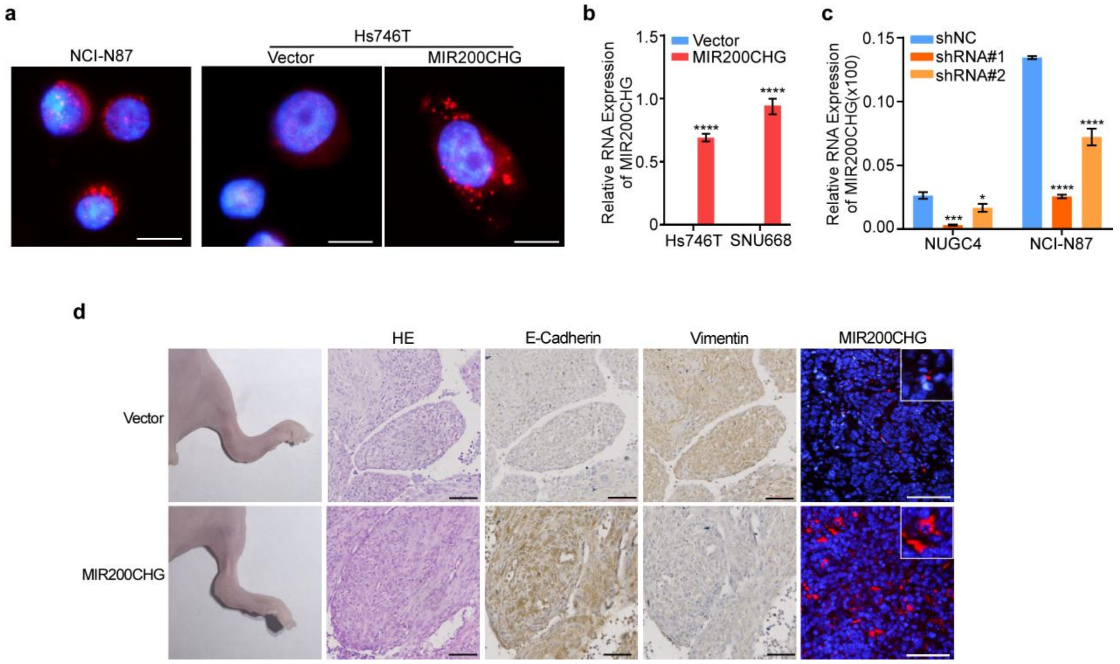

Supplementary Figure 5. Localization and expression of MIR200CHG in GC cell lines. a Representative image for MIR200CHG localization in NCI-N87 and Hs746T cells. Scale bar, 10 μm. **b-c** Ectopic expression of MIR200CHG in Hs746T, SNU668, NUGC4, and NCI-N87 GC cell lines, as detected by RT-qPCR. The RNA expression levels were normalized to those of GAPDH. Error bars represent the mean ± standard deviation calculated from three biologically independent samples. *P*-values were determined by two-sided Student's *t*-tests. **P* < 0.05, ***P* < 0.01, ****P* < 0.001, *****P* < 0.0001. **d** Representative image, HE staining, immunocytochemical analysis of E-cadherin and vimentin, and RNA FISH of MIR200CHG of the primary tumors of Hs746T Vector and MIR200CHG-overexpressed cell lines. Scale bar, 50 μm. The experiments were repeated three times independently with similar results **(a, d)**. Source data are provided as a Source Data file.

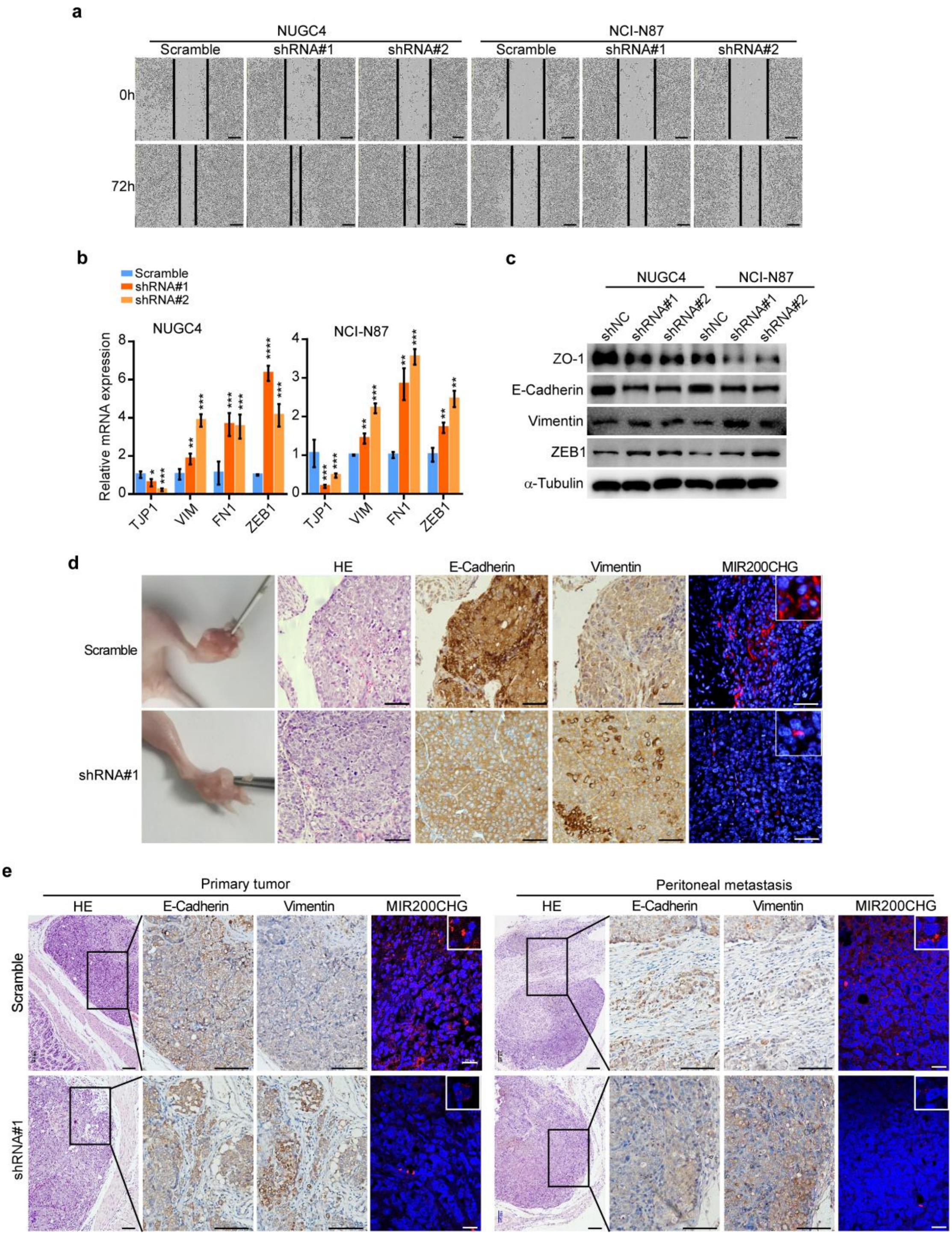

Supplementary Figure 6. MIR200CHG inhibits GC cell migration and tumor metastasis. a Wound healing analysis of NUGC4 and NCI-N87 cells for 72 h. Scale bar, 200 μm. The experiment was repeated of five biologically independent samples. **b** Expression of EMT relevant markers as determined by RT-qPCR. Each bar in bar plots represents the mean ± standard deviation of three biologically independent

samples. *P*-values were determined by two-sided Student's *t*-tests. **P* < 0.05, ***P* < 0.01, ****P* < 0.001, *****P* < 0.0001. **c** Protein expression of ZO-1, E-cadherin, vimentin, and ZEB1 in the indicated cells, as assessed by western blotting. **d-e** Representative image, HE staining, immunocytochemical analysis of E-cadherin and vimentin, and RNA FISH of MIR200CHG of the primary tumors of NCI-N87 Scramble and MIR200CHG-knockdown cell lines. Scale bar, 50 µm. The experiment was repeated three times independently with similar results **(c-e)**. Source data are provided as a Source Data file.

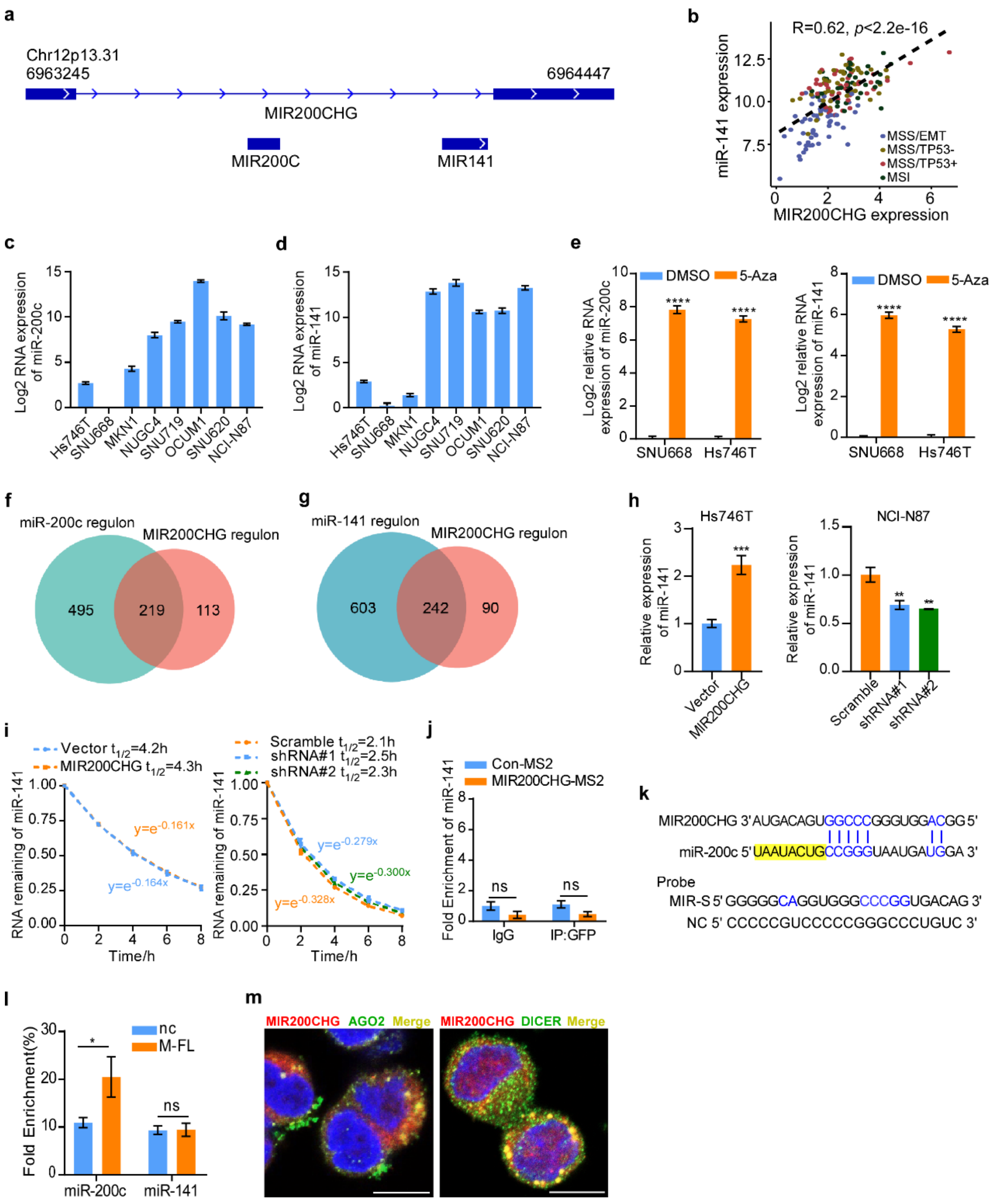

Supplementary Figure 7. Associations of MIR200CHG with miR-200c and miR-141. a The genomic localization of MIR200CHG, miR-200c, and miR-141. **b** The scatter plot shows a positive correlation between MIR200CHG expression and miR-141 expression in the TCGA cohort (*n* = 54 samples for MSS/EMT, *n* = 43 samples for MSS/TP53-, *n* = 44 samples for MSS/TP53+, *n* = 38 samples for MSI). *P*-value was based on two-sided Pearson's correlation test. **c** RT-qPCR analysis of miR-200c

expression in the MSS/EMT and non-MSS/EMT subtypes GC cell lines. **d** RT-qPCR analysis of miR-141 expression in the MSS/EMT and non-MSS/EMT subtypes GC cell lines. **e** 5-Aza (0.5 μM) treatment of the SNU668 and Hs746T cell lines revealed that a reduction in promoter methylation resulted in miR-200c and miR-141 re-expression. Venn diagrams show the overlaps (**f**) between the regulons of miR-200c and MIR200CHG, and (**g**) between the regulons of miR-141 and MIR200CHG. **h** The expression of miR-141 in MIR200CHG-overexpressed and MIR200CHG-knockdown cell lines was determined by RT-qPCR. **i** RT-qPCR analysis showed that MIR200CHG had no effect on the half-life of miR-141. **j** MS2-RIP and RT-qPCR analysis showed that MIR200CHG did not bind to miR-141 in HEK293T cells. **k** Sequence match between miR-200c and MIR200CHG (top) and the sequence of the RNA probe (MIR-S) containing the predicted miR-200c binding site and sequence of negative control probe (NC). The seed sequence was highlighted. **l** RNA-RNA pull down and RT-qPCR show that MIR200CHG directly binds to miR-200c but not miR-141. **m** IF-FISH demonstrates the co-localization of MIR200CHG and AGO2 or DICER. The experiment was repeated three times independently with similar result. Scale bar, 10 μm. Each bar in bar plots represents the mean ± standard deviation of three biologically independent samples **(c-e, h-j, l)**. *P*-values were determined by two-sided Student's *t*-tests **(e, h, j, l)**. *: $P < 0.05$, **: $P < 0.01$, ***: $P < 0.001$, ****: $P < 0.0001$, ns: not significant. Source data are provided as a Source Data file.

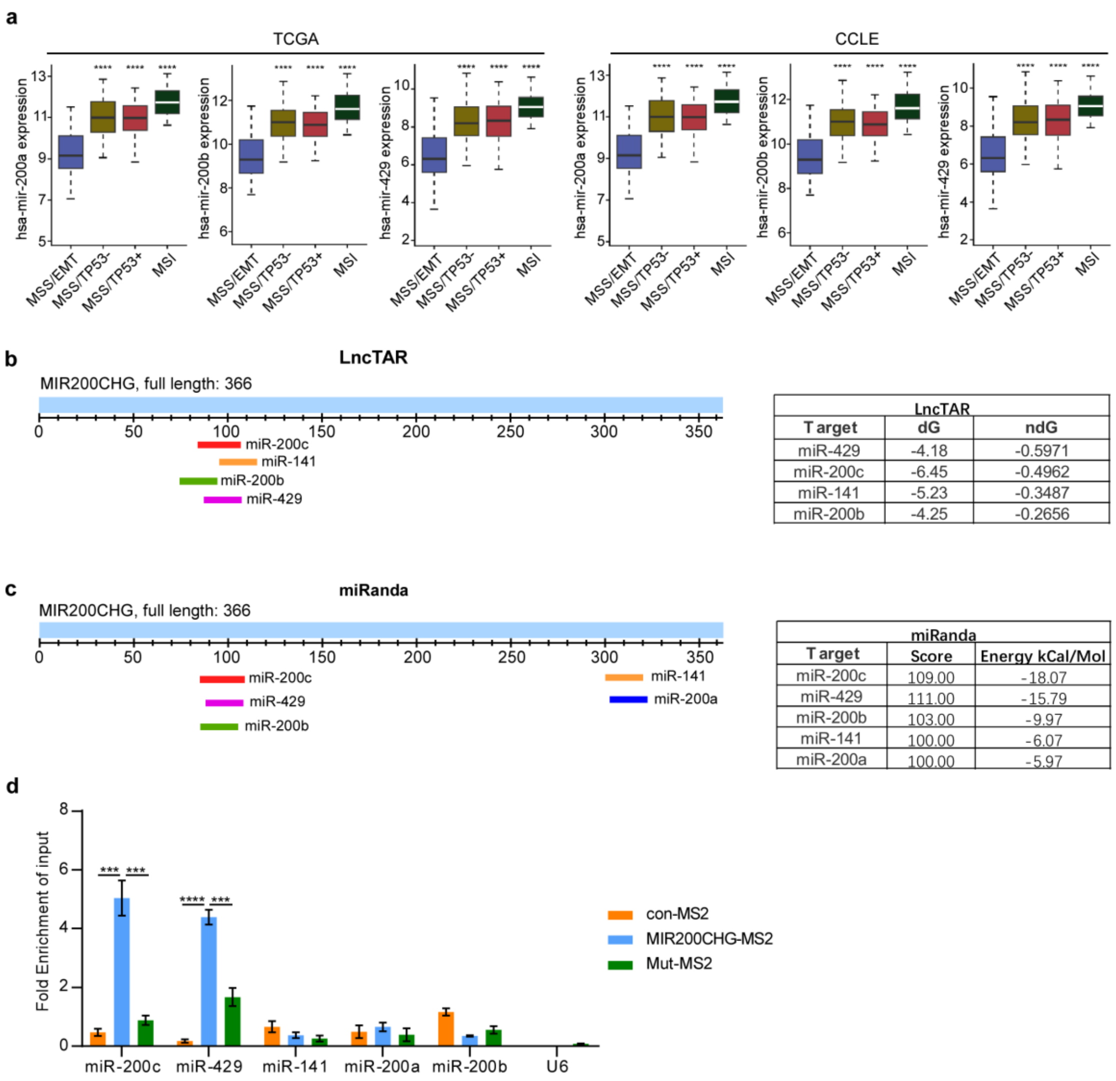

LncTAR		
Target	dG	ndG
miR-429	-4.18	-0.5971
miR-200c	-6.45	-0.4962
miR-141	-5.23	-0.3487
miR-200b	-4.25	-0.2656

miRanda		
Target	Score	Energy kCal/Mol
miR-200c	109.00	-18.07
miR-429	111.00	-15.79
miR-200b	103.00	-9.97
miR-141	100.00	-6.07
miR-200a	100.00	-5.97

Supplementary Figure 8. MIR200CHG binds to miR-200c and miR-429. a The boxplots showed significantly differential expression of miR-200a, miR-200b, and miR-429 between the MSS/EMT subtype and non-MSS/EMT subtypes in the TCGA cohort (*n* = 54 samples for MSS/EMT, *n* = 43 samples for MSS/TP53-, *n* = 44 samples for MSS/TP53+, *n* = 38 samples for MSI) and CCLE cohort (*n* = 37 cell lines, *n* = 6 for MSS/EMT, *n* = 18 for MSS/TP53-, *n* = 5 for MSS/TP53+, *n* = 8 for MSI). Boxes in the box-plots extend from the 25th to the 75th percentile and the lines indicate the median. The whiskers were drawn to the 5th and the 95th percentile. **b** LncTAR showed predicted binding sites of miR-200 paralogs on MIR200CHG. LncTAR utilizes a

variation on the standard "sliding" algorithm approach to calculate the binding free energy (ndG) and normalized binding free energy (ndG) to find the minimum free energy joint structure. **c** miRanda shows the predicted binding sites of miR-200 paralogs on MIR200CHG. The miRanda score and binding free energy of the miRNA and MIR200CHG were shown. **d** MS2-RIP and qRT-PCR analyses showed the interaction of MIR200CHG with miR-200 paralogs in HEK293T cells. Each bar in bar plots represents the mean ± standard deviation of three biologically independent samples. *P*-values were determined by two-sided Student's *t*-tests. ***: $P < 0.001$, ****: $P < 0.0001$. Source data are provided as a Source Data file.

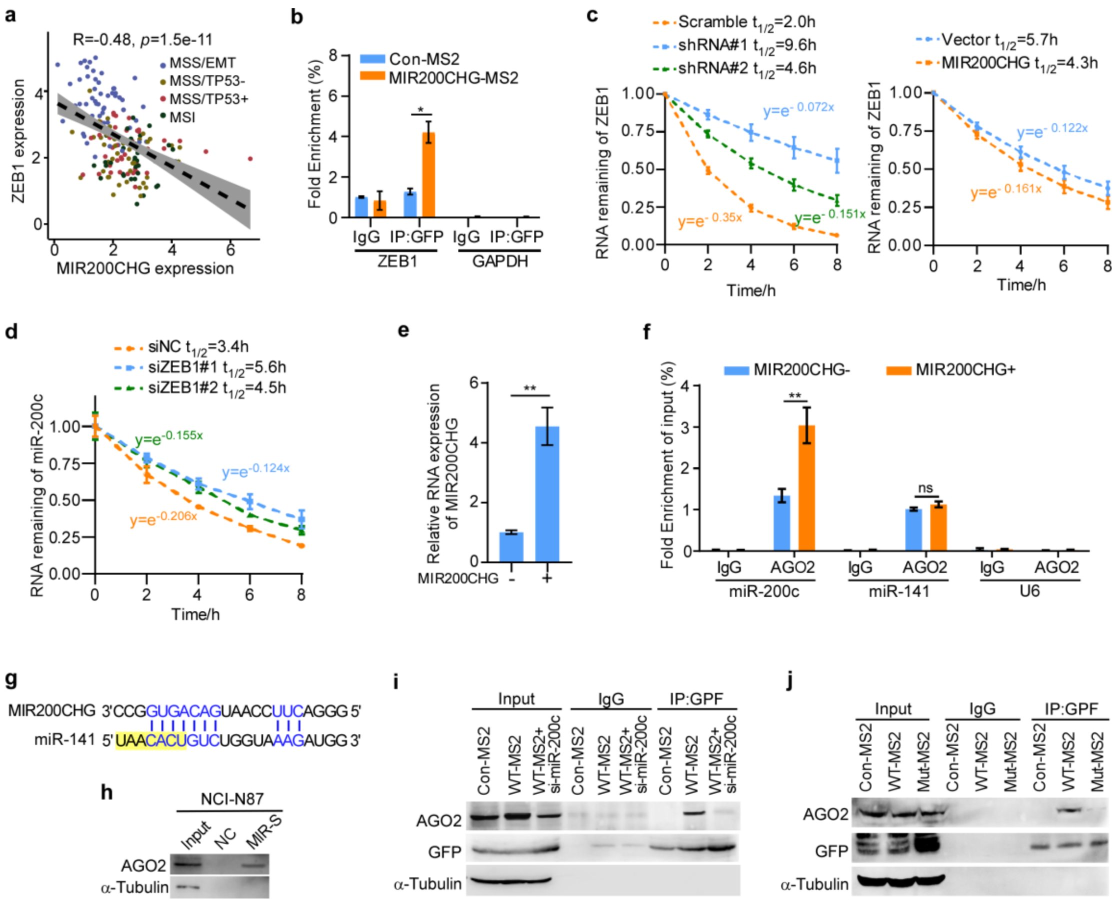

Supplementary Figure 9. MIR200CHG stabilizes AGO2 from proteasome degradation via competitively binding to the target of miR-200c. a The scatter plot shows the negative correlation between the MIR200CHG expression and ZEB1 expression in the TCGA-STAD cohort (n = 54 samples for MSS/EMT, n = 43 samples for MSS/TP53-, n = 44 samples for MSS/TP53+, n = 38 samples for MSI). P-value was based on two-sided Pearson's correlation test. **b** MS2-RIP and RT-qPCR analysis shows the interaction of miR200CHG with ZEB1 in 293T cells. **c** Extending ZEB1 mRNA half-life by silencing MIR200CHG and reducing ZEB1 mRNA half-life by overexpressing MIR200CHG. **d** Extending miR-200c RNA half-life by silencing ZEB1. **e-f** MIR200CHG overexpression plasmid was transfected into Hs746T cells. AGO2-RIP assay and RT-qPCR showed that more miR-200c occupied the same AGO2

protein when MIR200CHG was present. Each bar in bar plots represents the mean ± standard deviation of three biologically independent samples **(b-f)**. **g** The sequence match between miR-141 and MIR200CHG. The seed sequence was highlighted. **h** RNA-pull down and western blot showing the association of AGO2 protein with MIR200CHG in NCI-N87 cells. **i** HEK293T cells were transfected with siRNA targeting miR-200c in the presence of wild-type MIR200CHG (WT-MS2) followed by MS2-RIP assay. Western blotting shows the interaction of MIR200CHG with AGO2 protein. **j** HEK293T cells were transfected with wild-type MIR200CHG (WT-MS2) or MIR200CHG mutation (Mut-MS2) followed by MS2-RIP assay. Western blotting shows the interaction of MIR200CHG with AGO2 protein. The experiments were repeated three times independently with similar results **(h-j)**. *P*-values were determined by two-sided Student's *t*-tests **(b, e-f)**. *: $P < 0.05$, **: $P < 0.01$, ns: not significant. Source data are provided as a Source Data file.

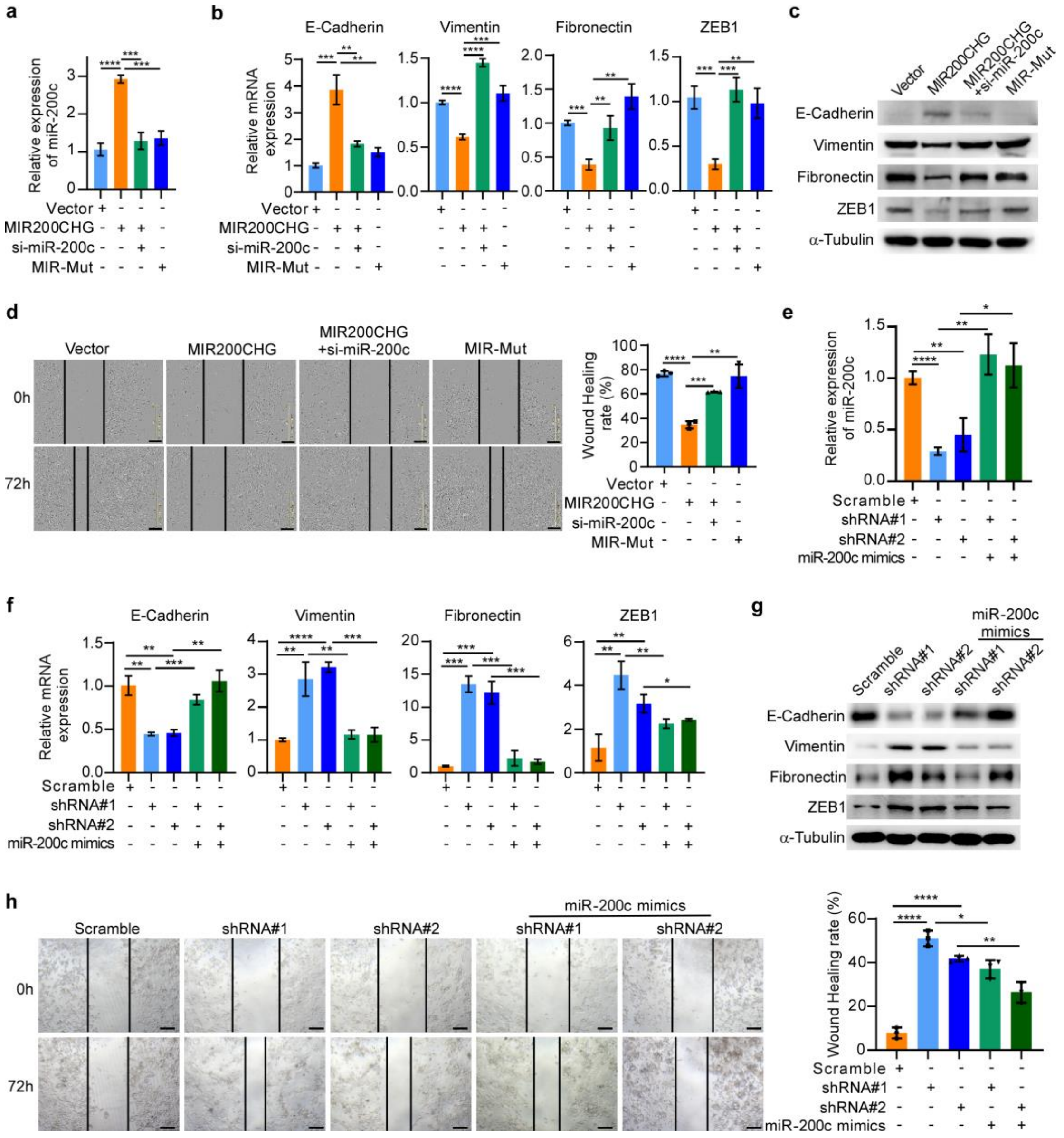

Supplementary Figure 10. MIR200CHG regulated EMT in a partially miR-200c-dependent manner. a miR-200c expression was detected by RT-qPCR in Hs746T cells. **b** The mRNA expression of fibronectin, E-cadherin, vimentin and ZEB1 were detected by RT-qPCR in Hs746T cells. **c** The protein expression of fibronectin, E-cadherin, vimentin and ZEB1 were detected by western blotting in Hs746T cells. **d** Wound healing analysis of Hs746T cells. **e** The expression of miR-200c was detected by RT-qPCR in NCI-N87 cells. **f** The mRNA expression of fibronectin, E-cadherin, vimentin and ZEB1 were detected by RT-qPCR in NCI-N87 cells. **g** The protein

expression of fibronectin, E-cadherin, vimentin and ZEB1 were detected by western blotting in NCI-N87 cells. **h** Wound healing analysis of NCI-N87 cells. **(a-d)** Hs746T cells were transfected with negative control siRNA or siRNA targeting miR-200c (si-miR-200c) in the presence or absence of a vector expressing MIR200CHG. **(e-h)** NCI-N87 cells were transfected with negative control siRNA or miR-200c mimics in the presence or absence of shRNA targeting MIR200CHG. Each bar in bar plots represents the mean ± standard deviation of three biologically independent samples **(a-b, d-f, h)**. *P*-values were determined by two-sided Student's *t*-tests **(a-b, d-f, h)**. *: $P < 0.05$, **: $P < 0.01$, ***: $P < 0.001$, ****: $P < 0.0001$. The experiments were repeated three times independently with similar results **(c, g)**. Source data are provided as a Source Data file.

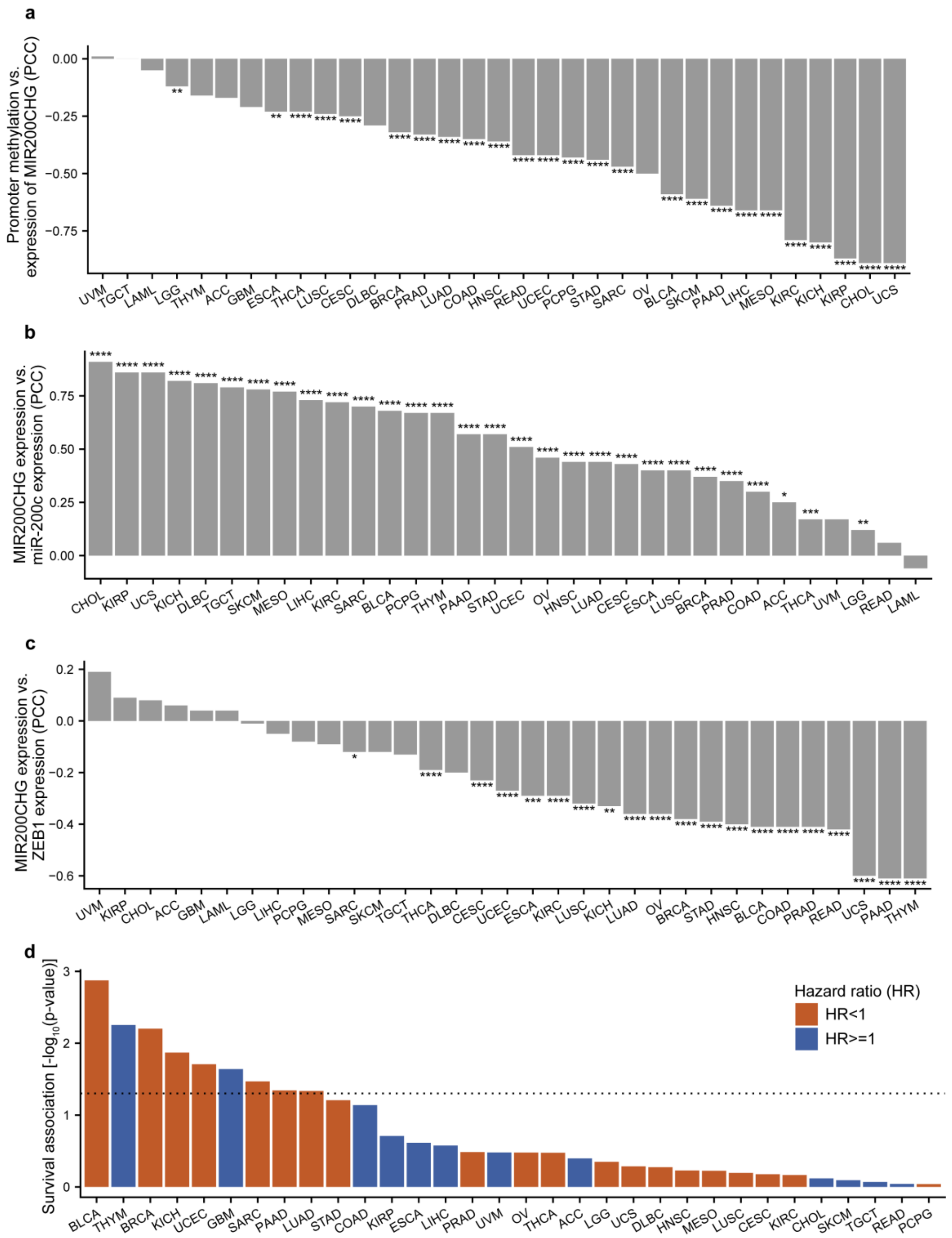

Supplementary Figure 11. Pan-cancer multi-omics analysis of MIR200CHG. a The bar plot shows the Pearson's correlation coefficients (PCCs) between the promoter methylation levels and expression levels of MIR200CHG across 33 cancers in TCGA. **b** The bar plot shows the PCCs between the expression levels of MIR200CHG and

miR-200c across 32 cancers (Glioblastoma is not shown due to the lack of miRNA expression data). **c** The bar plot shows the PCCs between the expression levels of MIR200CHG and ZEB1 across the 33 cancers. **d** The bar plot shows the association of MIR200CHG expression with overall survival, quantified by $-\log_{10}(P)$ derived from Univariate Cox regression analyses of MIR200CHG in 33 cancers. *P*-values were based on two-sided Pearson's correlation test **(a-c)**. *: $P < 0.05$, **: $P < 0.01$, ***: $P < 0.001$, ****: $P < 0.0001$.

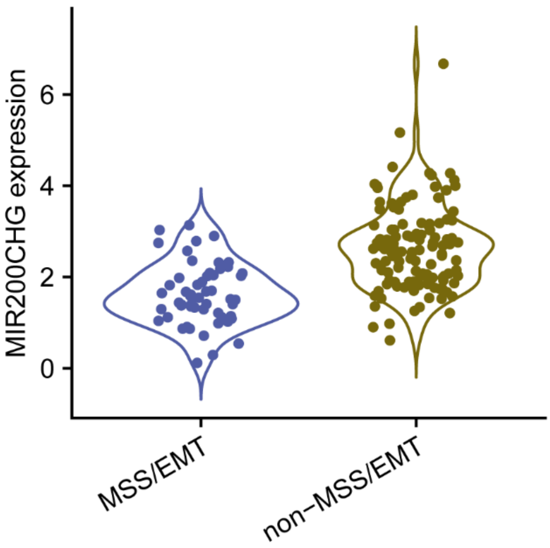

Supplementary Figure 12. The violin plot shows the MIR200CHG expression in MSS/EMT (*n* = 54 samples) and non-MSS/EMT (*n* = 125 samples) GC in the TCGA cohort.

Supplementary Table 1. Differentially expressed lncRNAs between each subtype and the others in the TCGA cohort.

Comparison	lncRNA	log2 fold change	Average expression	P-value	BH adjusted P-value
MSS/EMT vs. non-MSS/EMT	MIR100HG	1.62	1.36	6.46E-33	9.46E-29
	ZNF667-AS1	1.05	1.00	3.64E-31	1.33E-27
	LINC00578	1.20	1.05	5.64E-27	9.18E-24
	AP000892.3	1.58	1.23	9.60E-27	1.41E-23
	HAND2-AS1	1.36	0.62	5.74E-26	6.01E-23
	AC008808.2	1.02	0.79	6.23E-26	6.09E-23
	AC104083.1	1.62	2.75	2.11E-25	1.82E-22
	AP003071.4	1.20	0.79	4.40E-25	3.58E-22
	MBNL1-AS1	1.21	0.90	7.37E-25	5.68E-22
	ACTA2-AS1	1.15	0.97	2.70E-22	1.13E-19
	LINC00702	1.01	0.56	4.46E-22	1.64E-19
	PGM5-AS1	1.29	0.68	2.76E-21	8.97E-19
	AC053503.4	1.30	0.70	3.32E-20	9.53E-18
	CARMN	1.22	0.82	1.20E-19	2.98E-17
	AF001548.1	1.29	0.81	4.65E-19	1.02E-16
	AC093010.3	1.17	3.05	7.03E-19	1.45E-16
	FENDRR	1.23	1.63	3.25E-17	5.06E-15
	AL136084.3	1.21	1.75	5.28E-17	8.06E-15
	AF001548.3	1.02	0.53	1.11E-14	1.10E-12
	SERTAD4-AS1	1.17	1.61	2.25E-13	1.83E-11
	MIR200CHG	-1.01	2.33	5.62E-12	3.64E-10
	UCA1	-1.03	1.94	1.77E-04	2.00E-03
MSI vs. non-MSI	AC105460.1	1.95	0.80	1.57E-09	3.01E-06
	ELFN1-AS1	1.07	1.78	1.03E-05	1.63E-03
	AC005392.2	1.29	1.98	3.59E-05	3.68E-03
	LINC02381	-1.23	3.13	3.81E-05	3.83E-03
	H19	-1.26	3.18	3.20E-04	1.40E-02
MSS/TP53- vs. non MSS/TP53-			NA		
MSS/TP53+ vs. non MSS/TP53+			NA		

Note: *P*-values were determined by moderated two-sided *t*-test and adjusted for multiple testing.

Supplementary Table 2. Top differentially expressed lncRNAs between the MSS/EMT subtype and non-MSS/EMT subtypes in the TCGA cohort.

lncRNA	log2 fold change	Average expression	P-value	BH adjusted P-value
MIR100HG	1.62	1.36	6.84E-33	1.31E-29
LINC00578	1.20	1.05	5.91E-27	2.00E-24
AP000892.3	1.58	1.23	1.01E-26	3.26E-24
AC104083.1	1.62	2.75	2.20E-25	5.34E-23
AC093010.3	1.17	3.05	7.26E-19	5.56E-17
FENDRR	1.23	1.63	3.34E-17	2.05E-15
AL136084.3	1.21	1.75	5.43E-17	3.23E-15
SERTAD4-AS1	1.17	1.61	2.30E-13	8.63E-12
MIR200CHG	-1.01	2.33	5.73E-12	1.79E-10

Note: *P*-values were determined by moderated two-sided *t*-test and adjusted for multiple testing.

Supplementary Table 3. Sequence information used in this study.

siRNA/shRNA/probe name	Sequence (5'-3')
MIR200CHG shRNA-1	CCTTGGGAGCATGAAATAA
MIR200CHG shRNA-2	GCCTTTACAGCTGCAGCAA
MIR200CHG probe for FISH	ACATC+TTGCTGCAGC+TGTAAAG<br>CAAGGCGGGAAGACAA+TGGAGG<br>CAGAGGTTG+TTGGTCAGTAGTC
miR-200c mimics sense	AUUAUGACGGCCCAUUACUACCU
MIR200CHG-S (sense) probe for RNA-pulldown	GGGGGCAGGUGGGCCCGGUGACAG
MIR201CHG-AS (sense) probe for RNA-pulldown	CCCCCGUCCCCCGGGCCCUGUC
ZEB1-S (sense) probe for RNA-pulldown	UUUCUACCACAGUAUUAUAAUUUG
miR-200c siRNA	UCCAUCAUUACCCGGCAGUAUUA
siRNA negative control	CAGUACUUUUGUGUAGUACAAA

Supplementary Table 4. Sequences of primers used for RT-PCR and qRT-PCR in this study.

Primer name	Sequence (5'-3')
MIR200CHG Forward	CGTGGAATCTGGGGCCTTAA
MIR200CHG Reverse	ATCCAGAGGGGTGAAGGTCA
E-cadherin (CDH1) Forward	ATTTTTCCCTCGACACCCGAT
E-cadherin (CDH1) Reverse	TCCCAGGCGTAGACCAAGA
ZO-1 (TJP1) Forward	CAACATACAGTGACGCTTCACA
ZO-1 (TJP1) Reverse	CACTATTGACGTTTCCCCACTC
Vimentin (VIM) Forward	AGTCCACTGAGTACCGGAGAC
Vimentin (VIM) Reverse	CATTTCACGCATCTGGCGTTC
Fibronectin (FN1) Forward	AGGAAGCCGAGGTTTTAACTG
Fibronectin (FN1) Reverse	AGGACGCTCATAAGTGTCACC
ZEB1 Forward	TTACACCTTTGCATACAGAACCC
ZEB1 Reverse	TTTACGATTACACCCAGACTGC
miR-200c for RT-PCR	GTCGTATCCAGTGCAGGGTCCGAGGTATTCGCACTG GATACGACTCCATC
miR-200c for qRT-PCR	CGCGTAATACTGCCGGGTAAT
miR-141c RT-PCR	GTCGTATCCAGTGCAGGGTCCGAGGTATTCGCACTG GATACGACCCATCT
miR-141c for qRT-PCR	GCGCGTAACACTGTCTGGTAA
U6 for RT-PCR	AACGCTTCACGAATTTGCGT
U6 for qRT-PCR	F: CTCGCTTCGGCAGCACA<br>R: AACGCTTCACGAATTTGCGT
stem-loop for miRNA RT-PCR	GTCGTATCCAGTGCAGGGTCCGAGGTATTCGCACTG GATACGAC
Universal reverse primer for miRNA qRT-PCR	AGTGCAGGGTCCGAGGTATT
GAPDH Forward	GGAGCGAGATCCCTCCAAAAT
GAPDH Reverse	GGCTGTTGTCATACTTCTCATGG

Supplementary Table 5. Primary antibodies used in this study.

Antibody	Application	Company
E-cadherin (20874-1-AP)	1:5000 for WB, 1:200 for IF, 1:1000 for IHC	Proteintech
ZO-1 (66452-1-Ig)	1:1000 for WB, 1:500 for IF	Proteintech
Vimentin (10366-1-AP)	1:5000 for WB, 1:500 for IF, 1:2000 for IHC	Proteintech
Fibronectin (BS1644)	1:1000 for WB	Bioworld
ZEB1 (bs-4187R)	1:1000 for WB	Bioss
AGO2 (67934-1-Ig)	1:1000 for WB, 5ug for RIP, 1:200 for IF	Proteintech
DICER1(20567-1-AP)	1:100 for IF	Proteintech
GFP (66002-1-Ig)	1:1000 for WB, 5ug for RIP	Proteintech
α-Tubulin (RM2007L)	1:10000 for WB	RayAntibody
panCK (ab7753)	1 μg/ml for IHC	abcam
Goat anti-rabbit IgG (ab7090)	1:2000 for WB	abcam
Goat anti-mouse IgG (ab97040)	1:2000 for WB	abcam
Alexa Fluor 488 labeled anti-Rabbit (A11008)	1:500 for IF	Life Technologies
Alexa Fluor 488 labeled anti-Mouse (A11001)	1:500 for IF	Life Technologies
Alexa Fluor 594 labeled anti- Rabbit (A11012)	1:500 for IF	Life Technologies
Alexa Fluor 594 labeled anti-Mouse (A21203)	1:500 for IF	Life Technologies
Mouse IgG isotype control (66360-3-Ig)	5ug for RIP	Proteintech
