# Supplementary material for: LncRNA MIR200CHG inhibits EMT in gastric cancer by stabilizing miR-200c from target-directed miRNA degradation
Source: Nat Commun. 2023 Dec 8;14:8141. doi: 10.1038/s41467-023-43974-w (PMC10709323; doi:10.1038/s41467-023-43974-w)
Supplement: Supplementary file 3 — Description of Additional Supplementary Files [file 41467_2023_43974_MOESM3_ESM.pdf]

Description of Additional Supplementary Files

File name: Supplementary Data 1

Description: Top differentially expressed mRNAs (|log2FC| > 0.5, BH-adjusted $P < 1x10^{-5}$) between the MSS/EMT subtype and non-MSS/EMT subtypes in the TCGA cohort. *P*-values were determined by moderated two-sided *t*-tests and adjusted for multiple testing.

File name: Supplementary Data 2

Description: Demographic and clinical characteristics of gastric cancer patients.

File name: Supplementary Data 3

Description: Interacted molecules of MIR200CHG predicted by the ENCORI database.
