# Supplementary material for: LncRNA MIR200CHG inhibits EMT in gastric cancer by stabilizing miR-200c from target-directed miRNA degradation
Source: Nat Commun. 2023 Dec 8;14:8141. doi: 10.1038/s41467-023-43974-w (PMC10709323; doi:10.1038/s41467-023-43974-w)
Supplement: Supplementary file 7 — Reporting Summary [file 41467_2023_43974_MOESM7_ESM.pdf]

nature portfolio

Corresponding author(s): Xin Wang, Wenting Liao, Huilin Huang

Last updated by author(s): Oct 27, 2023

Reporting Summary

Nature Portfolio wishes to improve the reproducibility of the work that we publish. This form provides structure for consistency and transparency in reporting. For further information on Nature Portfolio policies, see our Editorial Policies and the Editorial Policy Checklist.

Statistics

For all statistical analyses, confirm that the following items are present in the figure legend, table legend, main text, or Methods section.

n/a	Confirmed	
☐	☒	The exact sample size (*n*) for each experimental group/condition, given as a discrete number and unit of measurement
☐	☒	A statement on whether measurements were taken from distinct samples or whether the same sample was measured repeatedly
☐	☒	The statistical test(s) used AND whether they are one- or two-sided<br>*Only common tests should be described solely by name; describe more complex techniques in the Methods section.*
☒	☐	A description of all covariates tested
☐	☒	A description of any assumptions or corrections, such as tests of normality and adjustment for multiple comparisons
☐	☒	A full description of the statistical parameters including central tendency (e.g. means) or other basic estimates (e.g. regression coefficient) AND variation (e.g. standard deviation) or associated estimates of uncertainty (e.g. confidence intervals)
☐	☒	For null hypothesis testing, the test statistic (e.g. *F*, *t*, *r*) with confidence intervals, effect sizes, degrees of freedom and *P* value noted<br>*Give P values as exact values whenever suitable.*
☒	☐	For Bayesian analysis, information on the choice of priors and Markov chain Monte Carlo settings
☒	☐	For hierarchical and complex designs, identification of the appropriate level for tests and full reporting of outcomes
☐	☒	Estimates of effect sizes (e.g. Cohen's *d*, Pearson's *r*), indicating how they were calculated

Our web collection on statistics for biologists contains articles on many of the points above.

Software and code

Policy information about availability of computer code

Data collection	R package ‘TCGAbiolinks’ (version 2.16.3) was used to retrieve data from The Cancer Genome Atlas (TCGA,https://portal.gdc.cancer.gov/).<br><br>R package ‘GEOquery’ (version 2.56.0) was used to download the GSE62254 (the ACRG cohort) and GSE15459 from Gene Expression Omnibus (GEO, https://www.ncbi.nlm.nih.gov/geo/).<br><br>All custom codes used in this study are available at https://github.com/CityUHK-CompBio/GC-MIR200CHG and have also been deposited on Zenodo: https://zenodo.org/records/10051102.<br><br>Bio-Rad CFX96 Real-Time System C1000 Cycler , IncuCyte ZOOM™ system, Nikon ECLIPSE Ti2, NIKON ECLIPSE 80i, Nikon ECLIPSE Ti2, NIKON ECLIPSE 80i, Tanon 5200 Multi were used to collect experimental data.
Data analysis	Differential expression analysis between each subtype of GC and the others was performed using the R package ‘limma’ (version 3.44.3).<br><br>GSEA was performed using the R package ‘HTSanalyzeR2’ (version 0.99.19).<br><br>LncRNA–mRNA regulatory network inference and master regulatory analysis was performed by R package ‘RTN’ (version 2.12.1).<br><br>The regulatory network was visualized using the R package ‘RedeR’ (version 1.36.0).<br><br>Univariate Cox proportional hazard regression analysis and survival analysis were performed using the R package ‘survival’ (version 3.2-3).

Fluorescence intensity was analyzed by ImageJ (version 1.37).

Statistical analysis for experimental assays was performed by GraphPad PRISM (version 8.0.2).

For manuscripts utilizing custom algorithms or software that are central to the research but not yet described in published literature, software must be made available to editors and reviewers. We strongly encourage code deposition in a community repository (e.g. GitHub). See the Nature Portfolio guidelines for submitting code & software for further information.

Data

Policy information about availability of data

All manuscripts must include a data availability statement. This statement should provide the following information, where applicable:

- Accession codes, unique identifiers, or web links for publicly available datasets
- A description of any restrictions on data availability
- For clinical datasets or third party data, please ensure that the statement adheres to our policy

The genome-wide mRNA and lncRNA expression profiles, 450K DNA methylation microarray data, miRNA expression data, and clinical information of 'TCGA-STAD' are available in The Cancer Genome Atlas (TCGA, https://portal.gdc.cancer.gov/). The latest lncRNA annotation for human is available from the GENCODE project (https://www.gencodegenes.org/human/). The gene expression profiles, miRNA expression profiles and DNA methylation profiles for 33 cancers are available in the TCGA database. Two independent gastric cancer gene expression datasets are available in Gene Expression Omnibus (GEO, https://www.ncbi.nlm.nih.gov/geo/) database under the accession code GSE62254 [https://www.ncbi.nlm.nih.gov/geo/query/acc.cgi?acc=GSE62254] and GSE15459 [https://www.ncbi.nlm.nih.gov/geo/query/acc.cgi?acc=GSE15459]. The gene expression profiles and drug sensitivity data of gastric cancer cell lines are available in the Cancer Cell Line Encyclopedia (CCLE, https://sites.broadinstitute.org/ccle/datasets). The remaining data are available within the article and supplementary information. Source data are available as a Source Data file. Source data are provided with this paper.

Human research participants

Policy information about studies involving human research participants and Sex and Gender in Research.

Reporting on sex and gender	*Use the terms sex (biological attribute) and gender (shaped by social and cultural circumstances) carefully in order to avoid confusing both terms. Indicate if findings apply to only one sex or gender; describe whether sex and gender were considered in study design whether sex and/or gender was determined based on self-reporting or assigned and methods used. Provide in the source data disaggregated sex and gender data where this information has been collected, and consent has been obtained for sharing of individual-level data; provide overall numbers in this Reporting Summary. Please state if this information has not been collected. Report sex- and gender-based analyses where performed, justify reasons for lack of sex- and gender-based analysis.*
Population characteristics	Of the 75 patients in the cohorts, 58 males and 16 females, 13 patients in T1/2 and 61 patients in T3/4, 25 patients in N0 and 50 patients in N1-3, 67 patients in M0 and 8 patients in M1.
Recruitment	*Describe how participants were recruited. Outline any potential self-selection bias or other biases that may be present and how these are likely to impact results.*
Ethics oversight	Informed consent by the patients and approval by the institutional review board.

Note that full information on the approval of the study protocol must also be provided in the manuscript.

Field-specific reporting

Please select the one below that is the best fit for your research. If you are not sure, read the appropriate sections before making your selection.

☒ Life sciences ☐ Behavioural & social sciences ☐ Ecological, evolutionary & environmental sciences

For a reference copy of the document with all sections, see nature.com/documents/nr-reporting-summary-flat.pdf

Life sciences study design

All studies must disclose on these points even when the disclosure is negative.

Sample size	No statistical methods were used to predetermine the experimental sample size. Sample sizes are indicated in the figure legends. Sample size was determined based on preliminary experiments that defined the adequate number of samples to consistently identify differences between groups.
Data exclusions	No data were excluded from the analyses.
Replication	Numbers of the experimental replication or the experiments that were performed independently for each specific result were indicated in the Figure Legends. All attempts at replication were successful.

Randomization | Allocation of samples is random.

Blinding | Blinding was not relevant to the study as it was retrospective in nature. The investigators were not blinded to outcome assessment, because results used were obtained using objective quantitative methods.

Reporting for specific materials, systems and methods

We require information from authors about some types of materials, experimental systems and methods used in many studies. Here, indicate whether each material, system or method listed is relevant to your study. If you are not sure if a list item applies to your research, read the appropriate section before selecting a response.

Materials & experimental systems

n/a	Involved in the study
☐	☒ Antibodies
☐	☒ Eukaryotic cell lines
☒	☐ Palaeontology and archaeology
☐	☒ Animals and other organisms
☐	☒ Clinical data
☒	☐ Dual use research of concern

Methods

n/a	Involved in the study
☒	☐ ChIP-seq
☒	☐ Flow cytometry
☒	☐ MRI-based neuroimaging

Antibodies

Antibodies used

The antibodies used for western blot:
Rabbit anti-E-cadherin (Proteintech, 20874-1-AP, 1:5000)
Mouse anti-ZO-1 (Proteintech, 66452-1-Ig, 1:1000)
Rabbit anti-Vimentin (Proteintech, 10366-1-AP, 1:5000)
Rabbit anti-Fibronectin (Bioworld, BS1644, 1:1000)
Rabbit anti-ZEB1 (Bioss, bs-4187R, 1:1000)
Mouse anti-AGO2 (Proteintech, 67934-1-Ig, 1:1000)
Mouse anti-GFP (Proteintech, 66002-1-Ig, 1:1000)
Mouse anti-α-Tubulin (RayAntibody, RM2007L, 1:10000)
Goat anti-rabbit immunoglobulin G (Abcam, ab7090, 1:2000)
Goat anti-mouse immunoglobulin G(Abcam, ab97040, 1:2000)
The antibodies used for Immunofluorescence:
Rabbit anti-E-cadherin (Proteintech, 20874-1-AP, 1:5000)
Mouse anti-ZO-1 (Proteintech, 66452-1-Ig, 1:1000)
Rabbit anti-Vimentin (Proteintech, 10366-1-AP, 1:5000)
Mouse anti-AGO2 (Proteintech, 67934-1-Ig, 1:200)
Rabbit anti- DICER1 (Proteintech, 20567-1-AP, 1:100)
Alexa Fluor 488 labeled anti-Rabbit (Life Technologies, A11008, 1:500)
Alexa Fluor 488 labeled anti-Mouse (Life Technologies, A11001, 1:500)
Alexa Fluor 594 labeled anti- Rabbit (Life Technologies, A11012, 1:500)
Alexa Fluor 594 labeled anti-Mouse (Life Technologies, A21203, 1:500)
The antibodies used for Immunohistochemistry:
Rabbit anti-E-cadherin (Proteintech, 20874-1-AP, 1:1000)
Rabbit anti-Vimentin (Proteintech, 10366-1-AP, 1:2000)
Mouse anti-pan Cytokeratin (abcam, ab7753, 1 μg/ml)
The antibodies used for RIP
Mouse anti-AGO2 (Proteintech, 67934-1-Ig, 5ug)
Mouse anti-GFP (Proteintech, 66002-1-Ig, 5ug)
Mouse IgG isotype control (Proteintech, 66360-3-Ig, 5ug)

Validation

The validation of primary antibodies were described by their manufacturers in the following websites:
The antibodies used for western blot:
Rabbit anti-E-cadherin (Proteintech, 20874-1-AP, 1:5000) https://www.ptgcn.com/products/E-cadherin-Antibody-20874-1-AP.htm
Mouse anti-ZO-1 (Proteintech, 66452-1-Ig, 1:1000) https://www.ptgcn.com/products/ZO1-Antibody-66452-1-Ig.htm
Rabbit anti-Vimentin (Proteintech, 10366-1-AP, 1:5000) https://www.ptgcn.com/products/VIM-Antibody-10366-1-AP.htm
Rabbit anti-Fibronectin (Bioworld, BS1644, 1:1000) https://www.antibodypedia.com/gene/3522/FN1/antibody/1560841/BS1644
Rabbit anti-ZEB1 (Bioss, bs-4187R, 1:1000) https://www.biossusa.com/products/bs-4187r
Mouse anti-AGO2 (Proteintech, 67934-1-Ig, 1:1000) https://www.ptglab.co.jp/products/AGO2-Antibody-67934-1-Ig.htm
Mouse anti-GFP (Proteintech, 66002-1-Ig, 1:1000) https://www.ptglab.co.jp/products/eGFP-Antibody-66002-1-Ig.htm
Mouse anti-α-Tubulin (RayAntibody, RM2007L, 1:10000) http://www.rayantibody.com/index.php?a=search&c=product&word=RM2007L
Goat anti-rabbit immunoglobulin G (Abcam, ab7090, 1:2000) https://www.abcam.cn/products/secondary-antibodies/goat-rabbit-igg-

hl-hrp-preadsorbed-ab7090.html
Goat anti-mouse immunoglobulin G(Abcam, ab97040, 1:2000) https://www.abcam.cn/products/secondary-antibodies/goat-mouse-igg-hl-hrp-preadsorbed-ab97040.html
The antibodies used for Immunofluorescence:
Rabbit anti-E-cadherin (Proteintech, 20874-1-AP, 1:5000) https://www.ptgcn.com/Products/E-cadherin-Antibody-20874-1-AP.htm
Mouse anti-ZO-1 (Proteintech, 66452-1-Ig, 1:1000) https://www.thermofisher.cn/cn/zh/antibody/product/ZO-1-Antibody-clone-1G4A1-Monoclonal/66452-1-IG
Rabbit anti-Vimentin (Proteintech, 10366-1-AP, 1:5000) https://www.ptglab.co.jp/products/VIM-Antibody-10366-1-AP.htm
Mouse anti-AGO2 (Proteintech, 67934-1-Ig, 1:200) https://www.ptglab.co.jp/products/AGO2-Antibody-67934-1-Ig.htm
Rabbit anti- DICER1 (Proteintech, 20567-1-AP, 1:100) https://www.ptglab.co.jp/Products/DICER1-Antibody-20567-1-AP.htm
Alexa Fluor 488 labeled anti-Rabbit (Life Technologies, A11008, 1:500) https://www.thermofisher.cn/cn/zh/antibody/product/Goat-anti-Rabbit-IgG-H-L-Cross-Adsorbed-Secondary-Antibody-Polyclonal/A-11008
Alexa Fluor 488 labeled anti-Mouse (Life Technologies, A11001, 1:500) https://www.thermofisher.cn/cn/zh/antibody/product/Goat-anti-Mouse-IgG-H-L-Cross-Adsorbed-Secondary-Antibody-Polyclonal/A-11001
Alexa Fluor 594 labeled anti- Rabbit (Life Technologies, A11012, 1:500) https://www.thermofisher.cn/cn/zh/antibody/product/Goat-anti-Rabbit-IgG-H-L-Cross-Adsorbed-Secondary-Antibody-Polyclonal/A-11012
Alexa Fluor 594 labeled anti-Mouse (Life Technologies, A21203, 1:500) https://www.thermofisher.cn/cn/zh/antibody/product/Donkey-anti-Mouse-IgG-H-L-Highly-Cross-Adsorbed-Secondary-Antibody-Polyclonal/A-21203
The antibodies used for Immunohistochemistry:
Rabbit anti-E-cadherin (Proteintech, 20874-1-AP, 1:1000) https://www.ptglab.co.jp/products/E-cadherin-Antibody-20874-1-AP.htm
Rabbit anti-Vimentin (Proteintech, 10366-1-AP, 1:2000) https://www.thermofisher.cn/cn/zh/antibody/product/Vimentin-Antibody-Polyclonal/10366-1-AP
Mouse anti-pan Cytokeratin (abcam, ab7753, 1 μg/ml) https://www.abcam.cn/products/primary-antibodies/pan-cytokeratin-antibody-c-11-ab7753.html
The antibodies used for RIP
Mouse anti-AGO2 (Proteintech, 67934-1-Ig, 5ug) https://www.ptglab.co.jp/products/AGO2-Antibody-67934-1-Ig.htm
Mouse anti-GFP (Proteintech, 66002-1-Ig, 5ug) https://www.ptgcn.com/products/GFP-tag-Antibody-HRP-66002.htm
Mouse IgG isotype control (Proteintech, 66360-3-Ig, 5ug) https://www.ptgcn.com/products/Mouse-IgG2b-isotype-control-Antibody-CL555-66360-3.htm

Eukaryotic cell lines

Policy information about cell lines and Sex and Gender in Research

Cell line source(s)	The GC cell lines SNU-668, MKN1, NUGC-4, NCI-N87, SNU-620, Hs746T, OCUM-1, and SNU-719 were purchased from ATCC (Manassas, VA, USA).<br>The GC cell lines SNU-668, MKN1, NUGC-4, NCI-N87, and SNU-620 were cultured in RPMI1640 medium supplemented with 10% fetal bovine serum (FBS).<br>Other cell lines – Hs746T, OCUM-1, and SNU-719 – were cultured in complete Dulbecco's modified Eagle medium supplemented with 10% FBS.<br>The human embryonic kidney cells (HEK293FT) were obtained from the Cell Bank of Shanghai Institutes of Biological Sciences (Shanghai, China) and cultured in Dulbecco's modified Eagle's medium (DMEM) with 10% FBS.<br>All of the cell lines were incubated at 37°C in a 5% CO2 atmosphere and were grown to 50%–80% confluence before the next passage or further experiments.
Authentication	All purchased cell lines were STR profiled.
Mycoplasma contamination	All used cell lines were mycoplasmen tested and had no mycoplasmen contamination.
Commonly misidentified lines<br>(See ICLAC register)	none

Animals and other research organisms

Policy information about studies involving animals; ARRIVE guidelines recommended for reporting animal research, and Sex and Gender in Research

Laboratory animals	BALB/c nude mice and NSG mice (4–5 weeks old, 18–20 g) were purchased from the Experimental Animal Center, Southern Medical University (Guangzhou, China), and were used to create the lymphatic metastasis models and Peritoneal metastasis model. Temperature (20–22) and humidity (40%–50%) on a 12-hour light/12-hour dark cycle.
Wild animals	none
Reporting on sex	none
Field-collected samples	none
Ethics oversight	All animal experiments were conducted after receiving approval from the Ethnics Committee of Southern Medical University (Guangzhou, China ,No. L2019023).

Note that full information on the approval of the study protocol must also be provided in the manuscript.

Clinical data

Policy information about clinical studies
All manuscripts should comply with the ICMJEguidelines for publication of clinical research and a completedCONSORT checklist must be included with all submissions.

Clinical trial registration	none
Study protocol	This was a retrospective collection of tissue samples from patients with gastric cancer in different clinical stages.
Data collection	Gastric cancer tissue chip was purchased from Shanghai OUTDO Biotechnology Co., LTD. (https://www.superchip.com.cn/index.html)
Outcomes	The primary endpoint was lymphatic metastasis.We collected the information about TMN stage of the GC patients, in which N0 stage was no lymph node metastasis and N1 to N3 stage was with lymph node metastasis.
